# Single species conservation as an umbrella for management of landscape threats

**DOI:** 10.1371/journal.pone.0209619

**Published:** 2019-01-09

**Authors:** Claire A. Runge, John C. Withey, David E. Naugle, Joseph E. Fargione, Kate J. Helmstedt, Ashley E. Larsen, Sebastian Martinuzzi, Jason D. Tack

**Affiliations:** 1 National Center for Ecological Analysis & Synthesis, University of California Santa Barbara, Santa Barbara, California, United States of America; 2 Graduate Program on the Environment, The Evergreen State College, Olympia, Washington, United States of America; 3 Wildlife Biology Program, University of Montana, Missoula, Montana, United States of America; 4 The Nature Conservancy, Minneapolis, United States of America; 5 Department of Environmental Science, Policy, and Management, University of California Berkeley, Berkley, California, United States of America; 6 Bren School of Environmental Science & Management, University of California, Santa Barbara, California, United States of America; 7 SILVIS Lab, Forest and Wildlife Ecology, University of Wisconsin-Madison, Madison, Wisconsin, United States of America; 8 United States Fish and Wildlife Service, Habitat and Population Evaluation Team, Missoula, Montana, United States of America; Sichuan University, CHINA

## Abstract

Single species conservation unites disparate partners for the conservation of one species. However, there are widespread concerns that single species conservation biases conservation efforts towards charismatic species at the expense of others. Here we investigate the extent to which sage grouse (*Centrocercus sp*.) conservation, the largest public-private conservation effort for a single species in the US, provides protections for other species from localized and landscape-scale threats. We compared the coverage provided by sage grouse Priority Areas for Conservation (PACs) to 81 sagebrush-associated vertebrate species distributions with potential coverage under multi-species conservation prioritization generated using the decision support tool Zonation. PACs. We found that the current PAC prioritization approach was not statistically different from a diversity-based prioritization approach and covers 23.3% of the landscape, and 24.8%, on average, of the habitat of the 81 species. The proportion of each species distribution at risk was lower inside PACs as compared to the region as a whole, even without management (land use change 30% lower, cheatgrass invasion 19% lower). Whether or not bias away from threat represents the most efficient use of conservation effort is a matter of considerable debate, though may be pragmatic in this landscape where capacity to address these threats is limited. The approach outlined here can be used to evaluate biological equitability of protections provided by flagship species in other settings.

## Introduction

Single-species conservation rose to popularity as an expedient, ecologically-based approach to spatial conservation prioritization. Ecologically, it was rooted in the notion that many species would benefit from conservation actions aimed at a single species. The early 2000s saw a proliferation of suggestions and criteria for choosing such ‘umbrella’ species [1,2] based on biological traits such as habitat use, mobility, and sensitivity to disturbance as well as logistical considerations such as data availability, policy mandates and funding availability. Over time it became evident that choosing sites for conservation action based on the occurrences or abundances of a single species did not always provide adequate ecological protection across species, taxa or ecological processes [2–4].

There is surprisingly little consensus in the literature on what, quantitatively, constitutes a ‘good’ umbrella species. Various metrics have been considered, from fixed targets (e.g. 10% overlap [5]) to comparison with the coverage provided by random sets of species [6]. In the analysis presented here, we extend the criteria in [6] and assess our umbrella species not just against a random naïve counterfactual, but by comparing its coverage to other site-selection strategies (e.g. multi-species prioritisation, threat risk).

Increases in computing power and the availability of conservation prioritization support tools, combined with the rise of remote sensing, citizen science datasets, and advances in modelling of species population dynamics and distribution, have made developing multi-species plans more tractable. These advances mean that conservation schemes, such as expansions of protected area networks, can now be designed to support a diversity of species or processes across multiple taxa [7,8]. Data and tools are readily available to support multi-species conservation plans, and multi-species and ecosystem-scale conservation is common practice in the marine realm (e.g. coral reef conservation) and is gaining traction on land through landscape-scale conservation cooperatives [9] and concepts such as the IUCN Red List for Ecosystems [10].

Yet, even as computational power has advanced, and multi-species prioritization is now commonplace in the academic realm, funding and policy still tend to favor single species conservation, whether through conservation initiatives designed around charismatic flagship species [11] (e.g. Sumatran orangutan) or within species management plans under national environmental legislation (e.g. Endangered Species Act in U.S.) Thus, it is fundamentally important to understand what is gained and lost by focusing on the conservation of a single species.

Despite potential drawbacks, such as focusing conservation on a single species at the cost of other, less charismatic species, work on flagship and charismatic species tells us that there is still a place for using one species to benefit many. They provide an avenue to achieve benefits for multiple species under the overarching aim of protecting one flagship species [12]. Flagship species attract more funding than non-flagship species [13], and may entice funding from sources that might not otherwise contribute to conservation [14]. Flagship species programs that connect with existing, positive, cultural associations can create emotional resonance and ownership among local communities [15, 16], generating intrinsic motivation that can contribute to conservation success [17].

Sage grouse are a successful example of flagship species conservation. In recent years sage grouse have attracted hundreds of millions of dollars of conservation investment to sagebrush habitat of the western US [18, 19]. While others have investigated the co-benefits of sage grouse conservation for one or a couple of species over limited geographic range [20–25], a comprehensive, range-wide assessment on the efficacy of sage grouse conservation as an umbrella for a multitude of sagebrush-dependent taxa is lacking. Here we use the flagship umbrella species concept to evaluate the ecological efficiency of that investment, exploring whether sage grouse conservation provides protection to sagebrush-associated vertebrate species. Sage grouse are iconic species of the western United States and are valued both for their unusual and charismatic breeding behavior and their value as a game species, in addition to being a cultural symbol of healthy rangelands. Sage grouse require large expanses of intact sagebrush habitat, which is one of the dominant biomes across North America, covering nearly 668,000 km^2^ of the western United States. Sagebrush is threatened by wildfire, cheatgrass (*Bromus tectorum*) invasion, pinyon-juniper encroachment, localized expansion of intensive cultivation and urban areas, and oil and gas drilling and mining development. These threats also impact the many species other than sage grouse that rely on sagebrush for at least some portion of their life history [26, 27].

We explore the extent to which sage grouse (which includes two species: Greater sage grouse *Centrocercus urophasianus* and Gunnison sage grouse *C*. *minimus*) conservation delivers co-benefits for the suite of 81 sage-associated vertebrate species and compare to that possible under multispecies prioritization approaches. We explore the bias in coverage across taxa and threats and identify the locations and species that might require conservation investment within the sagebrush biome outside the sage grouse umbrella.

## Materials and methods

Sage grouse conservation efforts have been spatially focused into Priority Areas for Conservation (PACs), which were developed with the primary objective of protecting key sage grouse populations via public lands policy and voluntary private lands conservation. PACs cover almost a quarter of the sagebrush biome on lands that are both publicly and privately owned. Public lands policy currently reduces the oil and gas footprints inside of PACs. The PACs have been used for targeting conservation. For example, the Sage Grouse Initiative (SGI), a private-lands initiative administered by the US Department of Agriculture’s Natural Resource Conservation Service, has enrolled 1,650 ranches in voluntary conservation programs. These ranches receive regulatory predictability under the federal Endangered Species Act such that, if the species is listed as a legally protected species, no additional conservation efforts will be required of them [28]. These PACs and conservation programs have so far negated the need to list the greater sage-grouse as a threatened or endangered species under the federal Endangered Species Act [29].

We evaluated the degree of coverage provided by Priority Conservation Areas (PACs) to the set of 81 vertebrate species that are strongly associated with sagebrush habitat in the western US (see section *Sagebrush species* below for the criteria we used for inclusion). We focused on PACs because they are current focal areas for conservation, encompassing 75% of sage grouse abundance, but covering only 23% of sagebrush and grasslands. To assess and improve the effectiveness of PACs for multispecies conservation, we first developed two systematic conservation prioritizations of protected areas: one designed to equitably cover the distributions of multiple species, and the other designed to retain rare species. Then, we compared these plans to the performance of PACs as protected areas, and identified which species are not well-represented by sage grouse conservation. We then used these species’ distributions to identify conservation priority locations outside of PACs and existing protected areas. PACs were designed on the basis of ecological criteria alone (sage grouse abundance), without consideration of sage grouse vulnerability to threats. Thus, we chose to ignore threat in these prioritisations to provide a similar basis for comparison for evaluation of the umbrella species concept. Species identity and their threats matter when deciding whether species fall under an umbrella or not (i.e. not enough just to count overlap) [30,31] and we evaluated the potential for PACs to protect species from threats. We estimated the risk to species inside and outside PACs from the main threats to sagebrush (i) cropland expansion, (ii) urban expansion, (iii) oil, gas and mining development (iv) forest encroachment and (v) cheatgrass invasion [32].

### Study area

**Fig 1:**
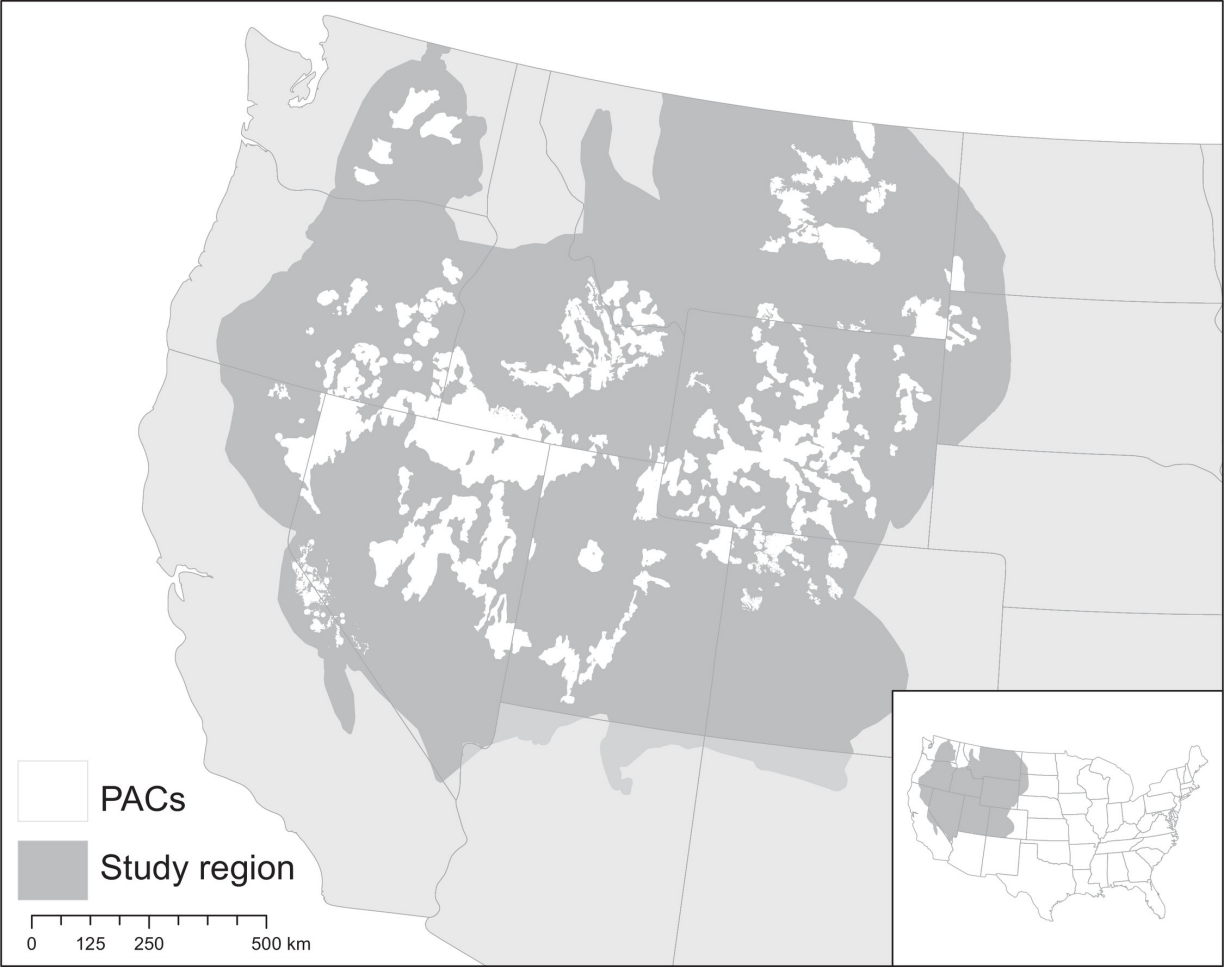
Map of the western US showing the boundaries of the sagebrush biome (study region) and Priority Areas for Sage Grouse Conservation (PACs), Albers equal area projection.

The study region was defined as sagebrush habitat across eleven states of the western US, incorporating California, Colorado, Idaho, Montana, Nevada, North Dakota, Oregon, South Dakota, Utah, Washington, and Wyoming (Fig 1). Sagebrush boundaries were defined by the region bounded by a polygon that included, within the continental US, 1) all existing sage grouse PACs and management zones [33], 2) the historic sage grouse species range [34], and 3) additional sagebrush cover (as shown by the Western United States Sagebrush Cover Raster [35]). Lands within the states of Nebraska, Arizona, and New Mexico were excluded from the study region, as sagebrush covers only a small fraction of their geography and sage grouse do not reside in these states. We excluded any cell classified as forest, water, cropland or developed land, using land use and land cover drawn from the 2011 National Land Cover Dataset [36]. Sagebrush, a name commonly used to describe shrubs in the genus *Artemisia*, dominates much of the landscape, with pockets of prairie grassland.

### Sagebrush species

We obtained species distribution maps for 81 sagebrush-associated vertebrate species found in the study region, across mammals, birds and reptiles (see S1 Table). Species were considered sage-associated based on 1) published sources [20, 37–42] and/or 2) an association with the NatureServe macrogroups “Western North America Tall Sage Shrubland & Steppe,” “Western North America Dwarf Sage Shrubland & Steppe,” and “Intermountain Dry Shrubland & Grassland” [43], as published by Lawler et al. [44]. Distribution maps for these species were drawn from three sources. Distribution models for 75 species were drawn from USGS Gap Analysis [45]. These maps are based on habitat associations described in published literature and integrate information on species associations with land cover, elevation, and hydrological characteristics. Information on the seasonal (summer, winter, year-round) distribution of species is included in these maps. These were supplemented with abundance models for three bird species: sage thrasher (*Oreoscoptes montanus*), sage sparrow (*Amphispiza bellii*), and Brewer’s sparrow (*Spizella breweri*) [23] and range maps for three large mammals: mule deer (*Odocoileus hemionus*), bighorn sheep (*Ovis canadensis*) and elk (*Cervus canadensis*) from game and fish agencies. Where seasonal distributions have been mapped for a species, we separately evaluated coverage and prioritization across summer (depending on the species, this included either summer-only or summer plus year-round distributions) or winter-only distributions (i.e., not including regions used year-round, to avoid double-counting those areas in the prioritization). There were 50 sage-associated species with year-round distributions, and 21 species with summer distributions only, represented by single conservation features. Another 10 species had both their summer and (spatially distinct) winter distributions within the study region represented by separate conservation features (i.e. 20 features for the 10 species) giving a total of 91 conservation features (excluding sage grouse).

Each of these distribution maps were evaluated for inclusion in our analyses based on the following thresholds: 1) the distribution overlapped ≥20% of our sagebrush biome (evaluated from species range map), or 2) >20% of the entire distribution was within our sagebrush biome polygon (Fig 1). This was intended to include both large-ranged species whose distribution overlaps significant portions of the sagebrush area, as well as smaller-ranged species that may overlap only a small part of the sagebrush area but depend on those habitats in a significant part of their range. Distribution maps were aggregated to 270m, taking the maximum value of the aggregated cells, and all analysis was conducted in ‘USA Contiguous Albers Equal Area Conic USGS version’ projection.

#### Overlap between PACs and species distributions

PACs cover 23.3% of shrubland and grassland in the study region. We calculated how well each species was represented within the area prioritized for sage grouse conservation by overlaying each species distribution with PAC boundaries. We compared this proportion with the proportion (23.3%) expected if protection were randomly distributed. PAC boundaries for greater sage grouse were drawn from [46], boundaries for Gunnison’s sage grouse from [33] and the PACs within Wyoming were updated with the 2016 dataset [47]. As the purpose of this study is to evaluate how to better protect sagebrush associated species, we ignored any part of a species distribution falling outside sagebrush habitat. The proportion of each species distribution falling outside this study area is included in S2 Table.

#### Comparing PACs to multi-species prioritization

Protected areas, defined as GAP Status Code of 1 or 2 (permanent protection from conversion of natural land cover), or IUCN class of Ia-IV (strictly protected, no extractive use) were classed as protected [48]. Together with PACs, they cover 35.3% of the grasslands and shrublands in this landscape. For each of the 81 sage-associated species, we compared the proportion of each species distribution that is currently held within existing protected areas and PACs with the proportion that could be protected in the same area under a multi-species prioritization. We used two alternative multi-species prioritization approaches. The *Prioritizing Richness* scenario prioritized local species richness and the *Prioritizing Rarity* scenario weighted rarer species more heavily.

We used the decision support tool Zonation version 4.0.0 [49] to generate multi-species landscape prioritizations. The output of Zonation is a hierarchical ranked map of the conservation value of a landscape, and a table listing the proportion of each species retained at each ranking. Landscape rank is identified by iteratively removing the least valuable cell according to a given objective function, accounting for generalized complementarity. Landscape ranking was determined on biological criteria alone (described below). Under both scenarios we accounted for the proportion of each species distribution already held in the existing protected area estate by setting protected areas to be the final cells removed from the landscape (ensuring they received the highest ranking).

We analysed the scenarios under two different objective functions. First, we considered an objective that favors vertebrate diversity-rich areas (Eq 1; *Prioritizing Richness*, which uses the Additive Benefit function (ABF) in Zonation). The marginal loss of biological value $\delta_{i}$ on removing cell *i* was defined as

$$\text{ABF}\;\delta_{i}=\frac{1}{c_{i}}\sum_{j}\left[{\left(q_{jn}\right)}^{0.25}-\;{\left(q_{jn-i}\right)}^{0.25}\right]$$ (1)

where *$q_{jn}$* is the proportion of feature *j* in the set of remaining cells *n*, $q_{jn-i}$ is the proportion of feature *j* in the set of remaining cells minus cell *i*, $q_{ji}$ is the proportion of the original full distribution of feature (species distribution) *j* located in cell *i*, and $c_{i}$ is the cost of adding cell *i* to the network.

Second, we considered an objective that prioritizes areas overlapping range-restricted species (Eq 2; *Prioritizing Rarity*, which uses the Core Area Zonation (CAZ) function in Zonation [50]). Here the marginal loss of vertebrate diversity value is defined as

$$\text{CAZ}\;\delta_{i}=\frac{1}{c_{i}}\max_{j}\frac{q_{ji}}{q_{jn}}$$ (2)

The *Prioritizing Richness* (ABF) function incorporates a benefit function describing the change in marginal value of habitat as the remaining area of habitat decreases that is comparable to a species-area curve. Under the *Prioritizing Rarity* (CAZ) function the marginal value is based on the most valuable feature in a cell, regardless of the value of that cell to other features.

We analysed two additional scenarios, *Prioritizing Richness outside PACs* and *Prioritizing Rarity outside PACs* to identify locations of biological importance not currently held within PACs or the existing protected area estate. This was achieved by setting PACs and protected areas to be the final cells removed from the landscape and thus preferentially retained in the conservation plan over areas not under protection. These scenarios used the objective functions described above.

In order to provide a consistent comparison with PACs, which are based solely on known sage grouse habitat, habitat ranking was based on biological criteria alone for all scenarios, rather than considering variable costs, such as cost of purchasing or placing an easement on private land or the opportunity cost of more restrictive policy on public lands. We evaluated performance of the multi-species prioritizations for sage grouse (using PAC boundaries as a surrogate for sage grouse distribution) post-prioritization. We used a paired t-test to determine if the mean difference in protection for any given species under current protection (PACs and protected areas) versus the multi-species scenarios (*Prioritise Richness* and *Prioritise Rarity*) is significantly different from zero.

#### Threat analysis

We calculated the spatial distribution of threats in this landscape and their overlap with PACs, by comparing the proportion of the landscape at risk inside PACs with the proportion of the study region at risk. We considered two threats: localized land use change (cropland expansion, urban expansion, oil, gas and mining development and forest encroachment), and cheatgrass invasion, a broad-scale threat. Maps of the spatial distribution of land use change threats in this landscape were drawn from four realistic and plausible future land use scenarios from USGS predictions of land use and land cover in year 2050 [51]. These maps use bio-geophysical and socioeconomic determinants under four IPCC development storylines (A1B, A2, B1, B2) to extrapolate land use and land cover (LULC) change from baseline 1992–2006 conditions. To clarify, these are maps of future land use not climate models. While these maps are often used as an input in climate change modelling, they are not predictions of land cover change in response to climate change. Maps of cheatgrass invasion risk were drawn from [52], and we considered areas classified as low resistance and resilience to be at high risk of invasion. Maps predictive of future renewable energy development were unavailable and thus this potential threat is not considered in this analysis, though evidence suggests that wind development in particular is more likely to occur in already disturbed landscapes (i.e. croplands) than is conventional energy development [53]. We overlaid these maps with species distributions and PAC boundaries to determine the proportion of each species range that is at risk within PACs and within the study region as a whole. We make the assumption that all sage-associated species are affected by direct loss of sagebrush to anthropogenic land uses and non-sagebrush land cover types.

Analysis was conducted in R version 3.4.0 using ‘raster’ package [54], and in ArcGIS version 10.4 [55]. R code is available at [56].

## Results

Sage grouse priority conservation areas (PACs) covered an average of 24.8% of the sagebrush distribution of each species. PACs provide better than random representation for 82% (67 of 81) of vertebrate species and 75% (68 of 91) of all conservation features evaluated (including winter distributions, excluding sage grouse; S3 Fig). The species whose ranges were best represented (>40% of total range) within PACs include dark kangaroo mouse (*Microdipodops megacephalus*), pygmy rabbit (*Brachylagus idahoensis*) and sage thrasher (*Oreoscoptes montanus)* (S1 Fig). Distributions of six special status species (i.e. species listed under the US ESA or as Near-Threatened or Endangered by the IUCN; see S1 Table) are prevalent in PACs. All but one of the carnivores, and three of four large hooved mammals of high conservation interest to the sporting public were also well represented within PACs (elk [*Cervus canadensis*], mule deer [*Odocoileus hemionus*] and pronghorn [*Antilocapra americana*]; S1 Fig). Sagebrush obligates and galliforms showed higher representation within PACs than other taxa (>40% coverage), and median protection across all groups was slightly higher than random, with the exception of wintering bird distributions (S2 Fig).

23 of the 91 conservation features considered have worse coverage within PACs than would be expected with random distribution of protected areas. More than half (13 of 23) of those poorly represented distributions are seasonal distributions of widespread raptors and small perching birds (see S2 Fig). Most migrate toward coastal areas or the desert Southwest. While these winter distributions overlap the edges of the sagebrush biome, they fall outside of the core sagebrush-steppe habitats where PACs are located (S2 Fig). PACs also provide worse-than-random protection for the full-year distributions of 10 species (S1 Fig). Species with worst coverage within PACs are pale kangaroo mouse (*Microdipodops pallidus*) and desert spiny lizard (*Sceloporus magister*). These are desert Southwest and Mexico-dwelling species whose distributions overlap the sagebrush boundary but have less than 5% overlap with PACs (see S3 Fig).

**Fig 2:**
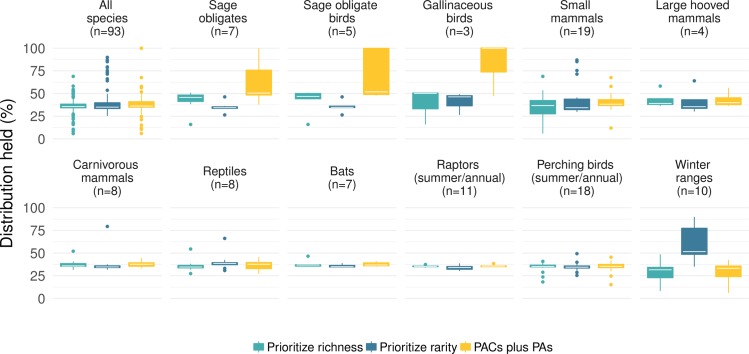
Comparison of the proportion of each species distribution (including sage grouse) currently held within Priority Areas for Sage Grouse Conservation (PACs plus PAs), and the proportion that could be held in an equivalent area prioritized across 81 sagebrush-associated species (91 species seasonal distributions, excluding greater and Gunnison’s sage grouse) under two objective functions (*Prioritize richness* & *Prioritize rarity*) using decision support tool Zonation. In all three scenarios we include the area already held in protected areas in the total for a species. The cross-species median coverage under each scenario is shown by a white line. The degree of overlap between these scenarios and PACs for the two sage grouse species was calculated after prioritization and these data points are included in this figure.

PACs and protected areas combined covered an average of 37.0% of the sagebrush distribution of each species. An equivalent area chosen using the *Prioritizing Rarity* function in Zonation (e.g. maximizing the conservation of rare species) provided additional coverage of 3.8% on average for each species (mean 40.9%, paired t-test df = 90 p 0.036, Figs 2 and S4). No significant difference was found between the mean percent coverage across the 81 species provided by PACs and protected areas and that possible under the *Prioritizing Richness* function (mean 36.0%, difference -0.9%, paired t-test df = 90 p 0.275). PACs conserve a slightly different suite of species to that protected using either the richness-based (ABF) or rarity-based (CAZ) objective function (Figs 2 and S3).

**Fig 3:**
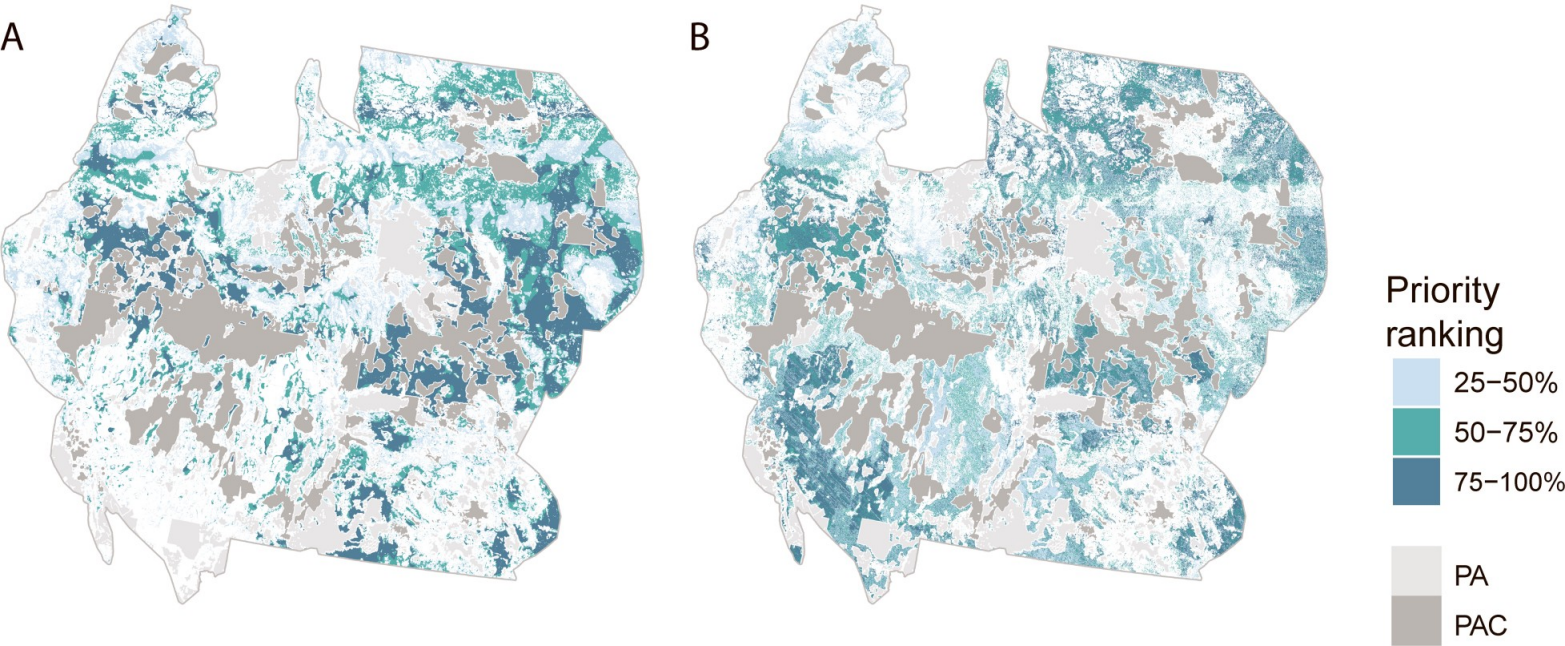
Conservation priority ranking of areas outside the existing protected area estate (PAs–light grey) and Priority Areas for Sage Grouse Conservation (PACs–dark grey) across the western US, Albers equal area projection. Ranking was based on biological criteria and generated by the decision support tool Zonation across a suite of 81 sagebrush-associated species, under two objective functions and accounting for complementarity (A) the *Prioritize richness outside PACs* scenario gives a higher ranking to locations that contain greater numbers of species, whereas (B) the *Prioritize rarity outside PACs* gives a higher ranking to locations containing rare or small-ranged species.

We found that broad-scale spatial priorities shifted depending on whether the objective favored species richness versus rarity (Fig 3A and 3B). For example, sagebrush and grasslands of Wyoming and Oregon and parts of the Dakotas were identified as high priorities for additional conservation under both *Prioritizing Richness outside PACs* and *Prioritizing Rarity outside PACs* scenarios. Lands surrounding existing PACs in Wyoming, Idaho and Oregon, as well as areas across the Dakotas ranked highly when the objective was to protect species richness (Fig 3A). In contrast, rarity emphasized desert regions of California and Nevada, and generated a more scattered solution overall (Fig 3B).

**Fig 4:**
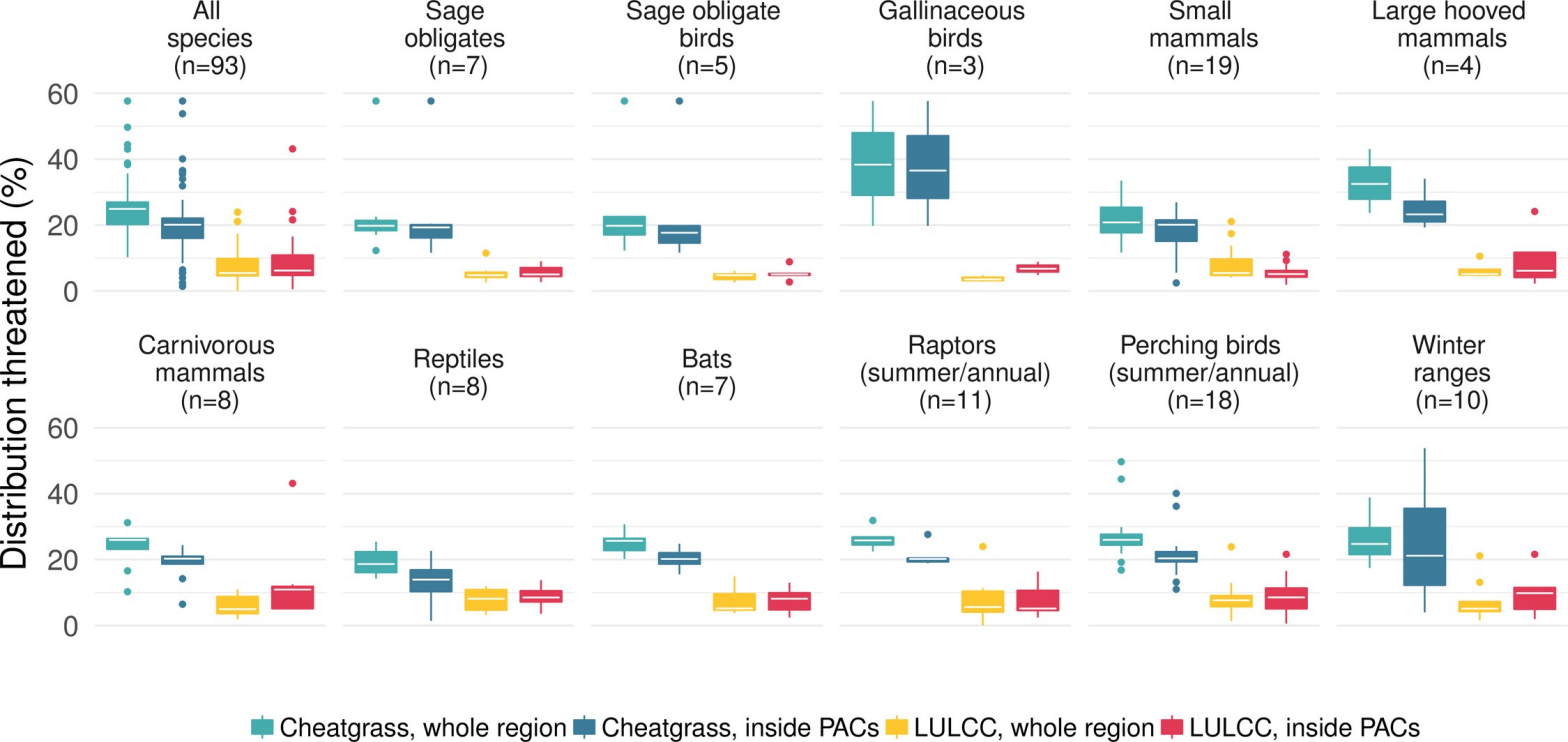
Proportion of 83 sagebrush-associated species (including sage grouse) ranges that are threatened by land use or land cover change (LULCC—urbanization, cropland conversion or forest expansion) by 2050 or by cheatgrass invasion. The proportion threatened across the whole study region (teal, yellow), including protected areas, is compared with that threatened within Priority Areas for Sage Grouse Conservation (PACs–blue, red). The cross-species median area threatened is shown by a white line.

We found that PACs tend to overlap places that, even without management, are at lower risk from future threats of development, cropland conversion and woodland encroachment, and cheatgrass invasion compared to areas outside PACs (Figs 4 and S5). Even ignoring the legal protections offered by PACs and PAs, the average proportion of each species distribution that is threatened by land-use change was around a third higher outside of PACs than inside (7.42% whole region, 5.16% in PACs, mean difference -0.68 CI-2.07–0.71%, df = 92, p 0.331), and sagebrush habitat was much less threatened overall inside PACs (5.2±0.4% is forecast to be impacted by land use change inside PACs, 11.5±0.8% across the whole biome, mean difference paired t-test 6.3% CI5.7–7.0, df = 3, p<0.0001). Similarly, across the study region, 23.9% of sagebrush is threatened by cheatgrass encroachment versus 20.3% inside PACs. Species distributions inside PACs are less likely to be at high risk of cheatgrass invasion even without management than their distributions across the study region as a whole (average inside 20.3%, average whole region 24.9, mean difference 4.6% CI3.5–5.8% df 91, p <0.0001).

## Discussion

Our findings demonstrate that investments in sage grouse as a flagship species (or in this case, two closely-related species) perform ecologically as well as a richness-based multispecies prioritization at protecting the 81 sagebrush-associated species we evaluated, and, for most species, provide a viable approach to ecosystem-scale sagebrush-steppe conservation [18]. Previous work indicates that sage grouse conservation can benefit other sagebrush-associated species. For example, in Wyoming, where sage grouse are most abundant, federal and state policy limiting energy development was supplemented with over $100US million in easements to conserve sagebrush steppe on private lands. Lease buyouts, energy policy and easements, all funded for sage grouse conservation, provide protection to 75% of migratory pathways for two iconic mule deer (*Odocoileus hemionus*) populations [22]. At the same time, proactively managing pinyon-juniper encroachment to improve sagebrush habitat quality for sage grouse [57] benefits other sagebrush-obligate birds, with 85% of restorations overlapping high songbird abundance landscapes [23] and Brewer’s sparrow (*Spizella breweri*) numbers increasing by half following targeted conifer removal [24].

The large spatial differences in the two prioritizations outside PACs indicate that choice of prioritization objective (and associated algorithm) has a large effect on the prioritization. Since PACs included a significant part of many species’ distributions and those distributions tend to be contiguous, it is perhaps not surprising that the *Prioritizing Richness outside PACs* approach identified many areas immediately surrounding PACs. In comparison, the *Prioritizing Rarity outside PACs* approach which was driven by the distributions of small-ranged species and priority areas were spread across the landscape (Fig 3). These differences in the areas identified as important outside of PACs emphasize that prioritization objective functions must be carefully selected to ensure they match conservation goals.

Whether or not conservation actions should be prioritized towards or away from areas at highest risk is a matter of ongoing debate. In part, it depends on the nature of species’ responses to threats and the conservation practitioner’s ability to ameliorate those threats [58]. In the western U.S., mineral rights are often severed and have different owners than surface rights; thus, many typical conservation actions, such as conservation easements, are inadequate to protect against mineral development. Species, such as sage grouse, that require large intact areas and are negatively impacted by low levels of development [59] will require protection of strongholds that are removed from the existing frontiers of development. PACs were designed on biological criteria alone, to represent the highest quality sage grouse habitat, rather than a systematic assessment of threats to sage grouse or feasibility of addressing those threats. The fact that vertebrate diversity is at lower risk from land use conversion and cheatgrass invasion inside of PACs is a fortunate artifact of sage grouse abundance.

Given the limited ability of conservation agencies to address the broader drivers of land use change, focusing efforts on maintaining high quality sagebrush in areas less likely to be threatened by land use conversion could be an option for species conservation. While the bias that we detected in the placement of PACs towards areas at low risk from direct loss may be seen to undermine the efficiency of a conservation strategy based on sage grouse PACs, the ability to control many of the threats to the sagebrush ecosystem in this landscape is limited by legal, cultural and practical constraints. For instance, though the threats of urbanization and cropland expansion are effectively prohibited on public lands (whether PAC or non-PAC), current options for directly restricting cropland conversion on private lands in the US, including PACs, are limited to voluntary strategies such as the acquisition of conservation easements. Some landholders are reluctant to enroll in these voluntary measures, which can limit their ability to adjust farm practices for future economic opportunities or climate uncertainties [60].

Similarly, we found PACs were less likely to be threatened by cheatgrass, an annual invasive grass that changes the productivity and fire dynamics of these ecosystems. This could be explained by the fact that the soil and moisture regimes that promote high-quality sagebrush habitat, and thus healthy sage grouse populations (key criteria for PACs), also make these areas more resistant to cheatgrass invasion, even in the absence of specific management. The science necessary to restore whole landscapes following conversions to invasive grasses is lacking [61]. Thus, targeting conservation investment, such as post-fire replanting of native grasses and shrubs, towards areas at lower threat from large-scale invasion may be more effective at maintaining sage grouse populations long term, though other species may benefit from the connectivity provided by retention of islands of habitat in areas at high risk of invasion.

As with any modeling effort, our study is subject to limitations. Firstly, we only assessed known threats to sagebrush rather than threats specific to each species, because science for many other species is rich in natural history but lacking in broad-scale threat assessment,. Additional information on how to best manage threats to other species will be needed in order to evaluate whether investments focused on PACs represent the most cost-efficient use of conservation effort in this landscape [30,31]. Climate change is likely to further exacerbate these threats and drive further changes to the distribution and abundance of species. However, projections of how and where climate might affect this socio-ecological system at the spatial scale necessary to quantitatively assess the effects of climate change on our analyses are currently lacking. We therefore decided not to include climate change in our analysis.

Secondly, species maps other than sage grouse and the three sage-associated birds represent their distributions but not their abundances and are subject to spatially unquantified uncertainty. Nonetheless, in a global meta-analysis evaluating effectiveness of the umbrella species concept, Branton and Richardson [4] report higher species abundances where umbrella species were present. Previous work reporting higher densities of sagebrush-obligate songbirds in PACs provides support for this trend in this landscape [23]. Species distributional data rarely comes with spatial information on uncertainty, and the datasets we used, though they represent the best available data, are no exception. Without this information it is not possible to quantify the uncertainty associated with the results presented here. However, previous research on the effect of uncertainty of species mapping on conservation prioritization indicates that omission and commission errors in species data have limited effect on the resulting prioritizations [62]).

Use of the umbrella species concept to emphasize the co-benefits for ecosystems, services or suites of species, rather than the gaps in protection under conservation actions for a given species may increase the perceived value of action to protect that species, increasing its attractiveness as a flagship species. This could allow access to additional resources (cross species funding) or increase investments provided by donors or governments [14]. Co-benefits that tie in with existing cultural values or political agendas may also increase the perceived value by local communities, and thus willingness to engage in conservation, a key criterion for flagship species [15, 16]. Game species such as sage grouse and bobwhite are beloved by hunting communities that invest heavily to conserve their heritage [63,64], making them good flagships [65]. Illuminating the gaps in flagship-species protection may provide impetus for complementary conservation actions for other species, including complementary flagship species [66], or for less charismatic species through systematic conservation prioritization, to fill those gaps [67]. This may help to avoid overlap in conservation efforts (to design complementary conservation efforts) or reduce conservation fatigue in a given community [68].

Our study revealed that sage grouse is a suitable surrogate for the majority of species identified as sage associates, though not all species benefited equally from sage grouse conservation. Consistent with [25] we found that highly localized species such as pale kangaroo mouse (*Microdipodops pallidus*), and those requiring specialized conservation actions such as black-footed ferret (*Mustela nigripes*) which exists only in intensively managed reintroduced populations, are unlikely to be adequately protected under the umbrella of flagship species. Species that happen to occur disproportionately on private lands may not be adequately covered by the limited protection provided by PACs. Consequently, flagship species conservation may need to be complemented by targeted and systematic investment [14, 69] to ensure equitable conservation across species. Finally, although our findings are encouraging for conservation in sagebrush habitat, they do not imply that sage grouse is indeed the optimal umbrella species in the region (i.e. the one that maximizes the collective abundance of species). Such action may require multi-scale approaches and comparisons of different potential species [70–72] which is out of the scope of this paper.

## Conclusions

Conservation has been built on decades of single-species focused plans and policies, but advances in conservation science and technology present opportunities to evaluate this paradigm. As we demonstrate here, while flagship species conservation can and does, buoy the presence of many other species, not all species will benefit equally. Alternative, or complementary, conservation prioritization approaches may be needed for range-limited species or those requiring specialized conservation actions to address threats to their persistence. The challenge lies in identifying conservation planning approaches that provide equitable protection across species while commanding the political and social support currently enjoyed by single-species conservation.

## Supporting information

S1 Table List of species. (PDF)

S2 Table Proportion of species distribution falling outside study region. (PDF)

S1 Fig Proportion of species distributions held within PACs. (PDF)

S2 Fig Proportion of species distributions held within PACs, by taxon. (PDF)

S3 Fig Proportional coverage under Zonation scenarios, by species. (PDF)

S4 Fig Species-area curves for the four Zonation scenarios. (PDF)

S5 Fig Proportion of distribution at risk, by species. (PDF)
