# Supplementary material for: Single species conservation as an umbrella for management of landscape threats
Source: PLoS One. 2019 Jan 9;14(1):e0209619. doi: 10.1371/journal.pone.0209619 (PMC6326495; doi:10.1371/journal.pone.0209619)
Supplement: S1 Table — (PDF) [file pone.0209619.s001.pdf]

S1 Table: List of species

Table S1: Species included in study. IUCN Red List status: CR=critically endangered, EN=endangered, VU=vulnerable, NT=near threatened. ESA threat status for 2016: NL=not listed, TH=threatened, EN=endangered

Scientific name	English common name	Taxon	Category	ESA threat status	IUCN Red list status
Ammodramus savannarum	Grasshopper Sparrow	bird	Perching bird	NL	LC
Amphispiza belli	Sage sparrow	bird	Sage obligate	NL	LC
Amphispiza bilineata	Black-throated sparrow	bird	Perching bird	NL	LC
Aquila chrysaetos	Golden eagle	bird	Raptors	NL	LC
Asio flammeus	Short-eared owl	bird	Raptors	NL	LC
Asio otus	Long-eared owl	bird	Raptors	NL	LC
Athene cunicularia	Burrowing owl	bird	Raptors	NL	LC
Bubo virginianus	Great horned owl	bird	Raptors	NL	LC
Buteo jamaicensis	Red tailed hawk	bird	Raptors	NL	LC
Buteo regalis	Ferruginous hawk	bird	Raptors	NL	LC
Buteo swainsoni	Swainson's hawk	bird	Raptors	NL	LC
Calamospiza melanocorys	Lark Bunting	bird	Perching bird	NL	LC
Centrocercus minimus	Gunnison sage-grouse	bird	Sage obligate, gallinaceous bird	TH	EN
Centrocercus urophasianus	Greater sage-grouse	bird	Sage obligate, gallinaceous bird	NL	NT
Charadrius montanus	Mountain plover	bird	Shorebird	NL	NT
Chondestes grammacus	Lark sparrow	bird	Perching bird	NL	LC
Circus cyaneus	Northern harrier	bird	Raptors	NL	LC
Empidonax wrightii	Gray flycatcher	bird	Perching bird	NL	LC
Eremophila alpestris	Horned lark	bird	Perching bird	TH	LC
Euphagus cyanocephalus	Brewer's blackbird	bird	Perching bird	NL	LC
Falco mexicanus	Prairie falcon	bird	Raptors	NL	LC
Falco peregrinus	Peregrine falcon	bird	Raptors	NL	LC
Lanius ludovicianus	Loggerhead shrike	bird	Perching bird	NL	LC
Numenius americanus	Long-billed curlew	bird	Shorebird	NL	LC
Oporornis tolmiei	MacGillvray's warbler	bird	Perching bird	NL	LC
Oreoscoptes montanus	Sage thrasher	bird	Sage obligate	NL	LC
Passerculus sandwichensis	Savannah sparrow	bird	Perching bird	NL	LC
Pipilo chlorurus	Green-tailed towhee	bird	Perching bird	NL	LC
Pooecetes gramineus	Vesper sparrow	bird	Perching bird	NL	LC
Salpinctes obsoletus	Rock wren	bird	Perching bird	NL	LC
Spizella breweri	Brewer's sparrow	bird	Sage obligate	NL	LC

Scientific name	English common name	Taxon	Category	ESA threat status	IUCN Red list status
Spizella passerina	Chipping sparrow	bird	Perching bird	NL	LC
Sturnella neglecta	Western meadowlark	bird	Perching bird	NL	LC
Tympanuchus phasianellus columbianus	Columbian sharp-tailed grouse	bird	Gallinaceous bird	NL	LC
Vermivora virginiae	Virginia's warbler	bird	Perching bird	NL	LC
Zenaida macroura	Mourning dove	bird	Perching bird	NL	LC
Zonotrichia leucophrys	White-crowned sparrow	bird	Perching birds	NL	LC
Antilocapra americana	Pronghorn	mammal	Large hooved mammal	NL[a]	LC
Antrozous pallidus	Pallid bat	mammal	Bat	NL	LC
Brachylagus idahoensis	Columbia Basin pygmy rabbit	mammal	Small mammal, sage obligate	TH	LC
Canis latrans	Coyote	mammal	Carnivorous mammal	NL	LC
Cervus canadensis	Elk	mammal	Large hooved mammal	NL	LC
Corynorhinus townsendii	Townsend's big-eared bat	mammal	Bat	NL[b]	LC
Cynomys leucurus	White-tailed prairie dog	mammal	Small mammal	NL	LC
Dipodomys microps	Chisel-toothed kangaroo rat	mammal	Small mammal	NL	LC
Dipodomys ordii	Ord's kangaroo rat	mammal	Small mammal	NL	LC
Eptesicus fuscus	Big brown bat	mammal	Bat	NL	LC
Euderma maculatum	Spotted bat	mammal	Bat	NL	LC
Lepus californicus	Black-tailed jackrabbit	mammal	Small mammal	NL	LC
Lepus townsendii	White-tailed jackrabbit	mammal	Small mammal	NL	LC
Lynx rufus	Bobcat	mammal	Carnivorous mammal	NL	LC
Microdipodops megacephalus	Dark kangaroo mouse	mammal	Small mammal	NL	LC
Microdipodops pallidus	Pale kangaroo mouse	mammal	Small mammal	NL	LC
Mustela frenata	Long-tailed weasel	mammal	Carnivorous mammal	NL	LC
Mustela nigripes	Black footed ferret	mammal	Carnivorous mammal	EN	EN
Myotis ciliolabrum	Western small-footed myotis	mammal	Bat	NL	LC
Myotis evotis	Long eared myotis	mammal	Bat	NL	LC
Myotis thysanodes	Fringed myotis	mammal	Bat	NL	LC
Odocoileus hemionus	Mule deer	mammal	Large hooved mammal	NL	LC
Ovis canadensis	Bighorn sheep	mammal	Large hooved mammal	EN[c]	LC
Perognathus longimembris	Little pocket mouse	mammal	Small mammal	NL	LC
Perognathus parvus	Great Basin pocket mouse	mammal	Small mammal	NL	LC

Scientific name	English common name	Taxon	Category	ESA threat status	IUCN Red list status
Peromyscus truei	Pinyon mouse	mammal	Small mammal	NL	LC
Puma concolor	Cougar	mammal	Carnivorous mammal	EN[d]	LC
Sorex merriami	Merriam's shrew	mammal	Small mammal	NL	LC
Sorex preblei	Preble's shrew	mammal	Small mammal	NL	LC
Spermophilus canus	Merriam's ground squirrel	mammal	Small mammal	NL	LC
Spermophilus elegans nevadensis	Wyoming ground squirrel	mammal	Small mammal	NL	LC
Spermophilus mollis	Piute ground squirrel	mammal	Small mammal	NL	LC
Spilogale gracilis	Western spotted skunk	mammal	Carnivorous mammal	NL	LC
Tamias dorsalis	Cliff chipmunk	mammal	Small mammal	NL	LC
Taxidea taxus	American badger	mammal	Carnivorous mammal	NL	LC
Thomomy bottae	Botta's pocket gopher	mammal	Small mammal	NL	LC
Thomomys talpoides	Northern pocket gopher	mammal	Small mammal	NL	LC
Vulpes macrotis	Kit fox	mammal	Carnivorous mammal	NL[e]	LC
Crotalus viridis concolor	Midget faded rattlesnake	Reptiles	Reptile	NL	LC
Crotaphytus bicinctores	Great Basin collared lizard	Reptile	Reptile	NL	LC
Gambelia wislizenii	Long-nosed leopard lizard	Reptile	Reptile	NL	LC
Phrynosoma douglasi	Short horned lizard	Reptile	Reptile	NL	LC
Phrynosoma platyrhinos	Desert horned lizard	Reptile	Reptile	NL	LC
Sceloporus graciosus	Common sagebrush lizard	Reptile	Reptile, sage obligate	NL	LC
Sceloporus magister	Desert spiny lizard	Reptile	Reptile	NL	LC
Uta stansburiana	Side blotched lizard	Reptile	Reptile	NL	LC

[a]subspecies *sonoriensis* (Sonoron pronghorn), is listed as threatened (ESA) and endangered (IUCN) but not present in study region.
[b]subspecies *virginianus* is listed as endangered (ESA) but not present in study region.
[c]subspecies *sierrae* only.
[d]proposed for delisting.
[e]subspecies *mutica* (San Joaquin kit fox), is listed as endangered (ESA) but not present in study region.
