# Supplementary material for: Single species conservation as an umbrella for management of landscape threats
Source: PLoS One. 2019 Jan 9;14(1):e0209619. doi: 10.1371/journal.pone.0209619 (PMC6326495; doi:10.1371/journal.pone.0209619)
Supplement: S2 Table — (PDF) [file pone.0209619.s002.pdf]

S2 Table: Proportion of species distribution falling outside study region

Table S2: Proportion of species distribution falling outside study region. Species seasonal ranges marked with an asterisk (*) were not included in the analysis as they did not meet our criteria for inclusion (see Methods).

English Common Name	Season	Percent of species range that falls within the sagebrush biome	Percent of sagebrush biome that species distribution overlaps
Brewer's blackbird	summer	99.0	33.5
Brewer's blackbird	winter	49.2	16.3
Black-throated sparrow	summer	32.9	25.2
Burrowing owl	summer	85.1	10.7
Burrowing owl*	winter	0.3	0.0
Chipping sparrow	summer	99.0	13.5
Chipping sparrow*	winter	1.4	0.8
White-crowned sparrow	summer	56.2	13.7
White-crowned sparrow	winter	74.1	19.2
Ferruginous hawk	summer	79.0	50.8
Ferruginous hawk	winter	21.6	13.3
Great horned owl	all year	99.7	7.3
Golden eagle	summer	98.1	4.5
Golden eagle	winter	99.7	4.6
Gray flycatcher	summer	46.3	74.4
Grasshopper Sparrow	summer	35.6	11.6
Grasshopper Sparrow*	winter	0.0	0.0
Green-tailed towhee	summer	67.3	70.9
Green-tailed towhee*	winter	1.5	1.2
Horned lark	summer	98.7	5.3
Horned lark	winter	99.7	7.1
Lark Bunting	summer	24.4	24.9
Lark sparrow	summer	88.5	28.2
Lark sparrow	winter	1.7	1.3
Long-billed curlew	summer	70.7	59.2
Long-billed curlew*	winter	0.0	0.0
Long-eared owl	summer	96.7	6.3
Long-eared owl	winter	98.4	6.2
Loggerhead shrike	summer	93.5	18.9
Loggerhead shrike	winter	38.0	12.3
MacGillvray's warbler	summer	65.3	44.2
Mourning dove	summer	99.7	16.0
Mountain plover	summer	26.0	60.0
Mountain plover*	winter	0.0	0.0
Northern harrier	summer	96.3	13.9
Northern harrier	winter	85.2	16.9
Peregrine falcon	summer	39.6	1.9

English Common Name	Season	Percent of species range that falls within the sagebrush biome	Percent of sagebrush biome that species distribution overlaps
Peregrine falcon	winter	7.9	0.3
Prairie falcon	summer	99.6	44.8
Prairie falcon	winter	99.7	34.0
Rock wren	summer	99.5	32.8
Rock wren*	winter	11.4	7.0
Brewer's sparrow	all year	97.8	54.1
Red tailed hawk	summer	99.7	11.5
Red tailed hawk[b]	winter	68.6	14.0
Savannah sparrow	summer	89.9	11.6
Savannah sparrow*	winter	6.1	2.5
Short-eared owl	summer	69.3	3.0
Short-eared owl	winter	99.7	5.9
Columbian sharp-tailed grouse	all year	33.6	9.5
Swainson's hawk	summer	99.7	25.6
Vesper sparrow	summer	93.1	26.0
Vesper sparrow*	winter	1.0	0.5
Virginia's warbler	summer	9.3	62.0
Western meadowlark	summer	99.7	26.5
Western meadowlark	winter	48.8	16.3
Sage sparrow	all year	45.3	76.8
Sage thrasher	all year	89.2	80.2
Bighorn sheep	all year	13.2	44.7
American badger	all year	99.9	20.2
Big brown bat	all year	99.9	13.6
Black footed ferret	all year	44.9	31.5
Bobcat	all year	100.0	19.2
Botta's pocket gopher	all year	17.5	21.6
Black-tailed jackrabbit	all year	53.4	22.3
Cliff chipmunk	all year	12.3	36.6
Cougar	all year	96.9	7.8
Coyote	all year	100.0	10.5
Chisel-toothed kangaroo rat	all year	18.9	90.3
Dark kangaroo mouse	all year	12.2	100.0
Fringed myotis	all year	57.7	30.6
Great Basin pocket mouse	all year	45.3	90.0
Kit fox	all year	25.0	26.3
Long eared myotis	all year	92.3	52.2
Little pocket mouse	all year	15.6	56.4
Long-tailed weasel	all year	98.4	14.3
Merriam's shrew	all year	80.2	80.4
Merriam's ground squirrel	all year	12.0	99.9

English Common Name	Season	Percent of species range that falls within the sagebrush biome	Percent of sagebrush biome that species distribution overlaps
Merriam's kangaroo rat*[a]	all year	4.9	6.6
Northern pocket gopher	all year	80.0	53.8
Ord's kangaroo rat	all year	69.9	36.8
Pallid bat	all year	61.4	25.8
Preble's shrew	all year	35.5	93.0
Piute ground squirrel	all year	22.5	99.1
Pinyon mouse	all year	35.9	46.1
Pale kangaroo mouse	all year	1.6	100.0
Pygmy rabbit	all year	27.7	100.0
Spotted bat	all year	57.2	49.7
Townsend's big-eared bat	all year	91.6	35.9
Mule deer	all year	100.0	28.2
Western small-footed myotis	all year	41.6	51.8
Western spotted skunk	all year	69.3	33.2
White-tailed jackrabbit	all year	89.5	46.0
White-tailed prairie dog	all year	14.6	98.6
Wyoming ground squirrel	all year	15.9	99.7
Pronghorn	all year	46.1	41.2
Elk	all year	52.2	45.5
Common sagebrush lizard	all year	63.9	79.7
Side blotched lizard	all year	38.4	34.2
Desert horned lizard	all year	27.8	65.1
Desert spiny lizard	all year	13.2	21.9
Great Basin collared lizard	all year	19.7	66.7
Long-nosed leopard lizard	all year	31.4	43.2
Midget faded rattlesnake	all year	35.8	32.6
Short horned lizard	all year	20.1	94.4
Gunnison sage-grouse	all year	0.5	100.0
Greater sage-grouse	all year	65.8	89.2

[a] Though Merriam’s kangaroo rat is widely listed in the literature as a sage-associated species, it did not meet our criteria for inclusion (see Methods).

[b] According to the GAP model (.tif) for red-tailed hawk, no habitat that is purely used for wintering (i.e. not also used in the summer) occurs in the study region, though both year round and summer habitat does. Our methodology aggregates summer and year round distributions into ‘summer’ distribution to avoid double counting species, and thus the wintering habitat was not analysed separately for this species.
