# Supplementary material for: Single species conservation as an umbrella for management of landscape threats
Source: PLoS One. 2019 Jan 9;14(1):e0209619. doi: 10.1371/journal.pone.0209619 (PMC6326495; doi:10.1371/journal.pone.0209619)
Supplement: S1 Fig — (PDF) [file pone.0209619.s003.pdf]

S1 Figure: Proportion of species distributions held within PACs

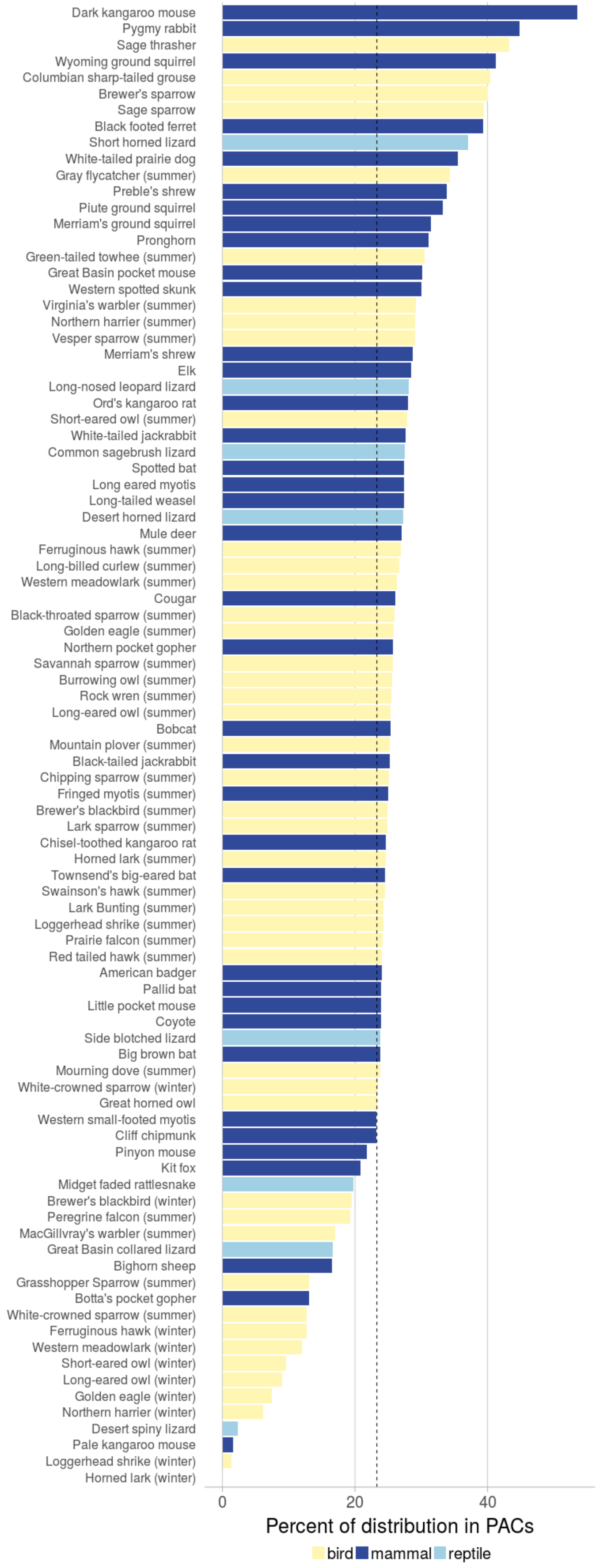

Figure S1: Area of species distribution (%) that occurs within Priority Areas for Sage Grouse Conservation (PACs), for 81 sagebrush-associated species of the western US. Taxon is indicated by colour (Reptile = pale blue, mammal=dark blue, bird=yellow). Only the part of each species distribution that occurs within the sagebrush biome held within a region bounded by eleven states of the western US is included in the calculation (see Methods for further details). The dashed vertical line indicates the proportion expected to be covered under random distribution of protection (23.3% of the landscape).
