# Supplementary material for: Single species conservation as an umbrella for management of landscape threats
Source: PLoS One. 2019 Jan 9;14(1):e0209619. doi: 10.1371/journal.pone.0209619 (PMC6326495; doi:10.1371/journal.pone.0209619)
Supplement: S2 Fig — (PDF) [file pone.0209619.s004.pdf]

**S2 Figure: Proportion of species distributions held within PACs, by taxon.**

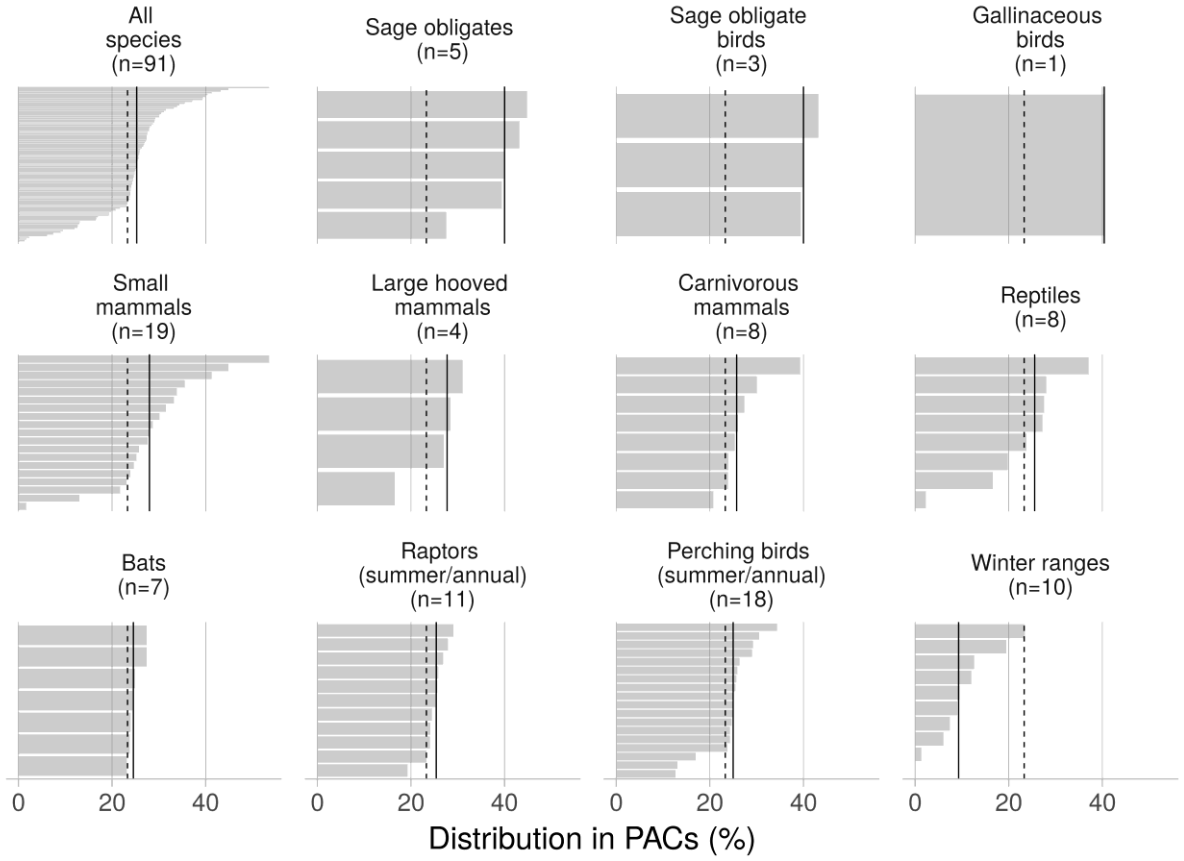


*Figure S2. Coverage provided by Priority Areas for Sage Grouse Conservation (PACs), for 81 sagebrush-associated species (excluding sage grouse) of the western US, where each bar represents a species seasonal distribution. Only the part of each species distribution that occurs within the sagebrush biome held within a region bounded by eleven states of the western US is included in the calculation (see Methods for further details). The dashed line indicates the proportion expected to be covered under random distribution of protection (23.3% of the landscape) and the cross-species median area held in PACs is shown as a solid black line.*
