# Supplementary material for: Single species conservation as an umbrella for management of landscape threats
Source: PLoS One. 2019 Jan 9;14(1):e0209619. doi: 10.1371/journal.pone.0209619 (PMC6326495; doi:10.1371/journal.pone.0209619)
Supplement: S3 Fig — (PDF) [file pone.0209619.s005.pdf]

S3 Figure: Proportional coverage under ZONATION scenarios, by species.

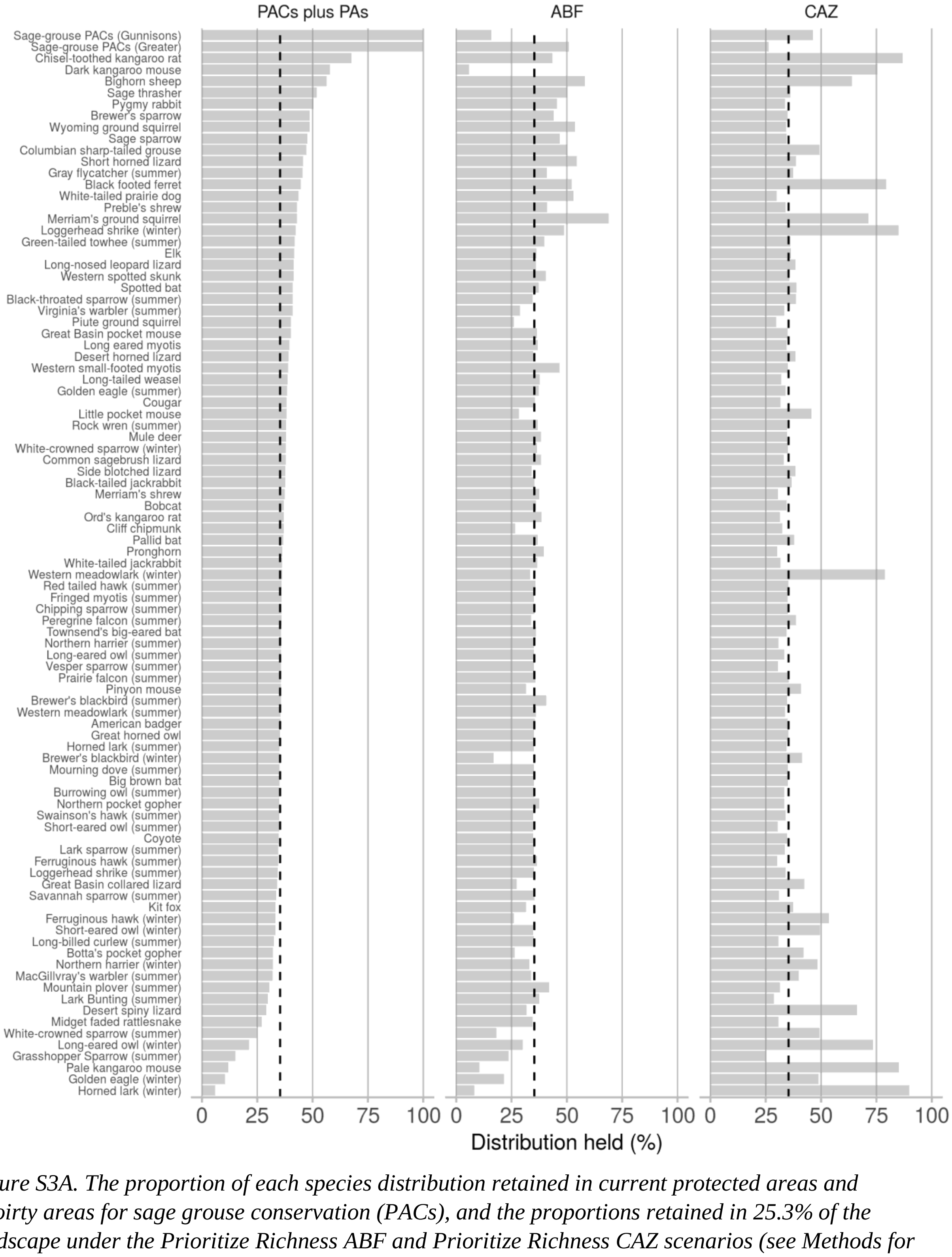

Figure S3A. The proportion of each species distribution retained in current protected areas and prioirty areas for sage grouse conservation (PACs), and the proportions retained in 25.3% of the landscape under the Prioritize Richness ABF and Prioritize Richness CAZ scenarios (see Methods for details).

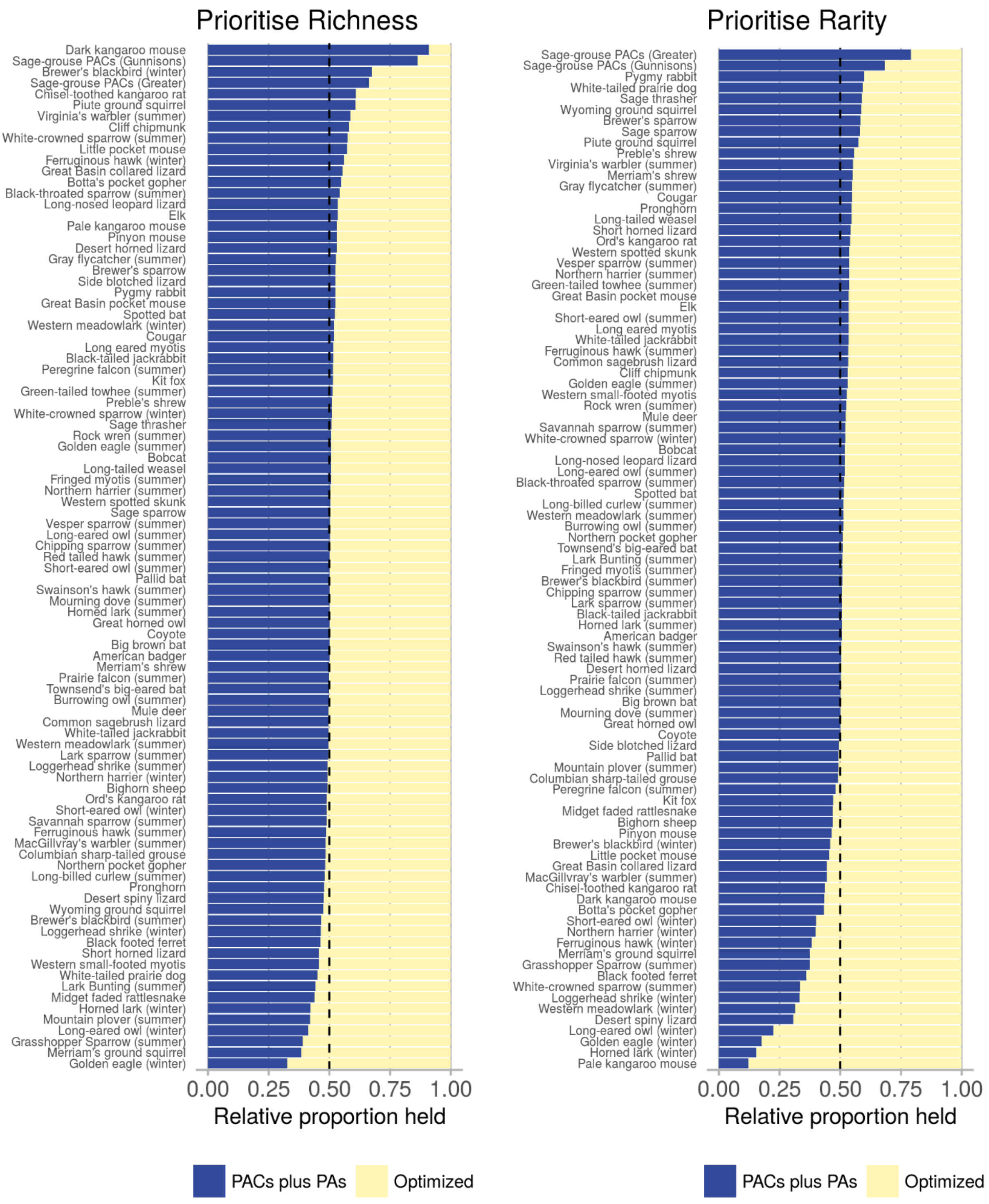

*Figure S3B. Comparison between the proportion of each species distribution held within protected areas and Priority Areas for Sage Grouse Conservation (PACs plus PAs - blue), with the proportion that could be held in an equivalent area prioritized across 81 sagebrush-associated species (*Prioritize Richness - ABF *and* Prioritize Rarity - CAZ *- yellow). The vertical dashed line indicates the point at which PACs perform equally to the prioritizations.*
