# Supplementary material for: Single species conservation as an umbrella for management of landscape threats
Source: PLoS One. 2019 Jan 9;14(1):e0209619. doi: 10.1371/journal.pone.0209619 (PMC6326495; doi:10.1371/journal.pone.0209619)
Supplement: S4 Fig — (PDF) [file pone.0209619.s006.pdf]

S4 Figure: Species-area curves for the four ZONATION scenarios

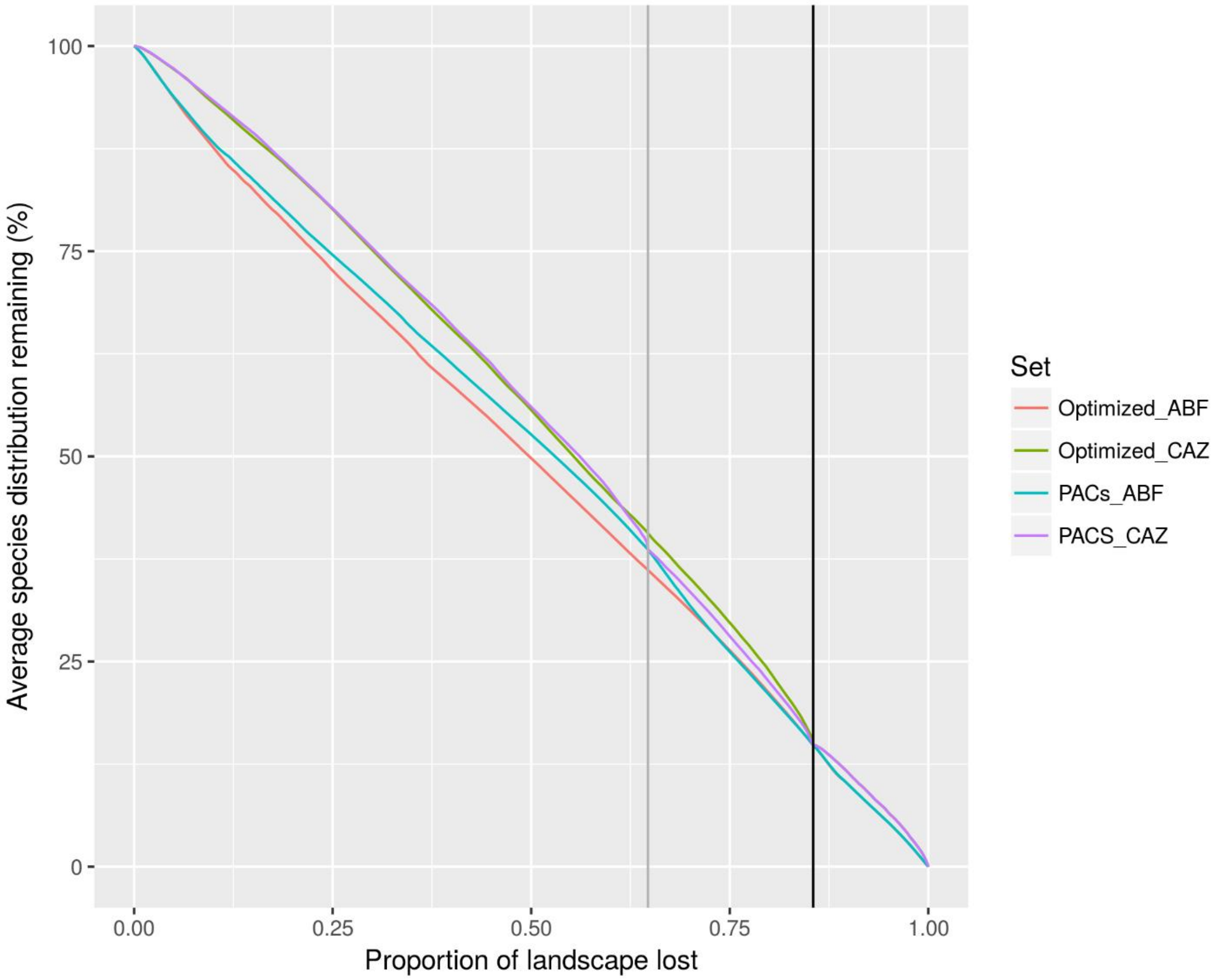

Figure S4: Species-area curves for the four ZONATION scenarios. The coloured lines are averaged across 81 sage-associated species. The black vertical line represents the area held in current protected areas and the grey vertical line represents the additional area equivalent to that held in Priority Areas for Sage Grouse Conservation (PACs). See Methods for details on scenario formulation.
