# Supplementary material for: Single species conservation as an umbrella for management of landscape threats
Source: PLoS One. 2019 Jan 9;14(1):e0209619. doi: 10.1371/journal.pone.0209619 (PMC6326495; doi:10.1371/journal.pone.0209619)
Supplement: S5 Fig — (PDF) [file pone.0209619.s007.pdf]

S5 Figure: Proportion of distribution at risk, by species

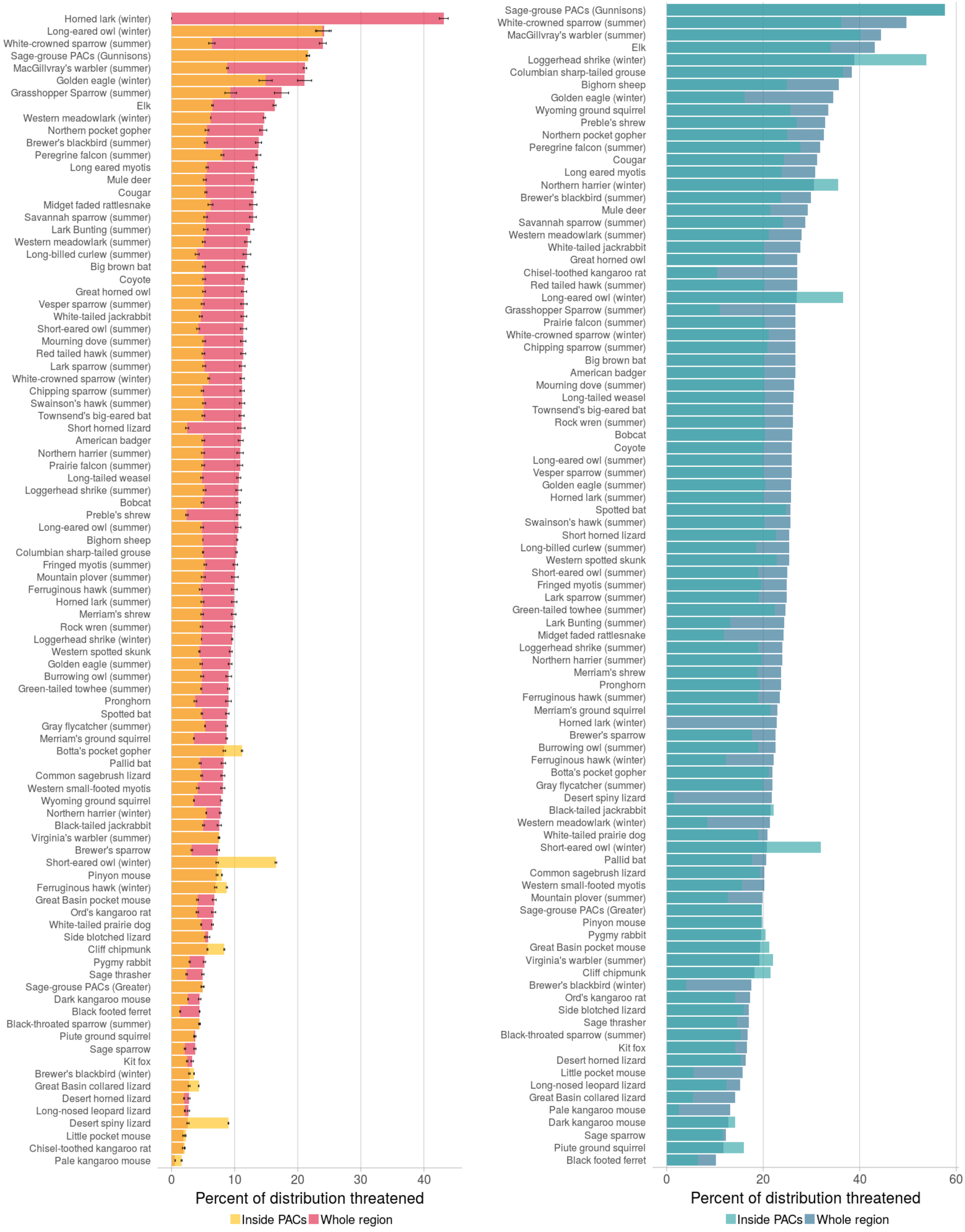

Figure S5. Proportion of 83 sagebrush-associated species (including two sage grouse species) distributions (92 conservation features, including seasonal distributions) across the western US that is threatened by (a) urbanization, cropland conversion or forest expansion by 2050 (b) cheatgrass invasion. The proportion threatened that is held within Priority Areas for Sage Grouse Conservation (PACs) is compared with that threatened in the whole study region. Error bars in (a) show standard errors across four equally plausible future scenarios.
